# Optimizing hip MRI: enhancing image quality and elevating inter-observer consistency using deep learning-powered reconstruction

**DOI:** 10.1186/s12880-025-01554-y

**Published:** 2025-01-13

**Authors:** Yimeng Kang, Wenjing Li, Qingqing Lv, Qiuying Tao, Jieping Sun, Jinghan Dang, Xiaoyu Niu, Zijun Liu, Shujian Li, Zanxia Zhang, Kaiyu Wang, Baohong Wen, Jingliang Cheng, Yong Zhang, Weijian Wang

**Affiliations:** 1 Department of Magnetic Resonance Imaging, The First Affiliated Hospital, Zhengzhou University, Zhengzhou, 450052 China; 2 Department of Radiology, The Third Affiliated , Zhengzhou University, Zhengzhou, 450052 China; 3 MR Research China, GE Healthcare, Beijing, China

**Keywords:** Hip Joint, Deep learning, MRI, Image quality, Diagnostic performance

## Abstract

**Background:**

Conventional hip joint MRI scans necessitate lengthy scan durations, posing challenges for patient comfort and clinical efficiency. Previously, accelerated imaging techniques were constrained by a trade-off between noise and resolution. Leveraging deep learning-based reconstruction (DLR) holds the potential to mitigate scan time without compromising image quality.

**Methods:**

We enrolled a cohort of sixty patients who underwent DL-MRI, conventional MRI, and No-DL MRI examinations to evaluate image quality. Key metrics considered in the assessment included scan duration, overall image quality, quantitative assessments of Relative Signal-to-Noise Ratio (rSNR), Relative Contrast-to-Noise Ratio (rCNR), and diagnostic efficacy. Two experienced radiologists independently assessed image quality using a 5-point scale (5 indicating the highest quality). To gauge interobserver agreement for the assessed pathologies across image sets, we employed weighted kappa statistics. Additionally, the Wilcoxon signed rank test was employed to compare image quality and quantitative rSNR and rCNR measurements.

**Results:**

Scan time was significantly reduced with DL-MRI and represented an approximate 66.5% reduction. DL-MRI consistently exhibited superior image quality in both coronal T2WI and axial T2WI when compared to both conventional MRI (*p* < 0.01) and No-DL-MRI (*p* < 0.01). Interobserver agreement was robust, with kappa values exceeding 0.735. For rSNR data, coronal fat-saturated(FS) T2WI and axial FS T2WI in DL-MRI consistently outperformed No-DL-MRI, with statistical significance (*p* < 0.01) observed in all cases. Similarly, rCNR data revealed significant improvements (*p* < 0.01) in coronal FS T2WI of DL-MRI when compared to No-DL-MRI. Importantly, our findings indicated that DL-MRI demonstrated diagnostic performance comparable to conventional MRI.

**Conclusion:**

Integrating deep learning-based reconstruction methods into standard clinical workflows has the potential to the promise of accelerating image acquisition, enhancing image clarity, and increasing patient throughput, thereby optimizing diagnostic efficiency.

**Trial registration:**

Retrospectively registered.

**Supplementary Information:**

The online version contains supplementary material available at 10.1186/s12880-025-01554-y.

## Key points

Deep learning based reconstruction Magnetic Resonance Imaging (DL-MRI) not only expedites scan times but also significantly enhances imaging quality.

**Supplementary Information:**

The online version contains supplementary material available at 10.1186/s12880-025-01554-y.

## Background

Magnetic Resonance Imaging (MRI) stands as the recommended choice for assessing the hip joint, thanks to its remarkable ability to provide detailed representations of soft-tissue structures. In the context of contemporary healthcare, the significance of exploring tissues beyond bone in hip pain research has garnered increasing recognition [1, 2]. MRI distinguishes itself by its capacity to directly portray all anatomical elements within the joint, making it particularly well-suited for scrutinizing non-osteochondral structures, given its innate proficiency in accentuating soft tissues [3]. MRI’s unique capability lies in its capacity to assess the joint as a unified organ, yielding a level of detail unparalleled by any other imaging modality. However, despite its widespread utilization in the evaluation of musculoskeletal conditions, MRI comes with a drawback: the necessity for lengthy scan durations to capture high-quality images. This extended scan time contributes to elevated costs and may inadvertently induce anxiety in some patients. Furthermore, patient discomfort can trigger involuntary movements, introducing artifacts into the imaging process [4, 5].

In the past, various image acquisition acceleration techniques, such as partial Fourier encoding [6], parallel imaging [7–9], and incoherent sparse sampling of data points, have successfully shortened MRI scan durations. However, these techniques have reached a performance limit, where conventional reconstruction methods struggle to adequately reconstruct the undersampled data. Recognizing the need to transcend the limitations of conventional MRI acquisition and reconstruction techniques, there has been a recent surge in the application and development of deep convolutional neural network (CNN) models [10, 11] in the field of MRI. Notably, a commercially available deep learning-based reconstruction (DLR) pipeline, known as AIR Recon DL by GE Healthcare [12, 13], has emerged as a significant advancement. It’s important to emphasize that DLR doesn’t merely enhance undersampled information but rather enhances low signal-to-noise images acquired in shorter intervals through its powerful denoising capabilities.

To date, the application of DL-accelerated imaging techniques has extended to various musculoskeletal areas, including the spine, shoulder, and knee [4]. For instance, Hahn et al. [14] conducted a comparative study involving standard shoulder MRI sequences and accelerated sequences both with and without DLR. Their findings not only demonstrated a reduction in scanning time with DLR but also highlighted its ability to achieve comparable diagnostic performance. However, it’s worth noting that, thus far, there has been a limited number of studies specifically addressing the application of DLR in the context of hip pain assessment.

Against this backdrop, the primary objective of our study was to conduct a comprehensive comparison. We aimed to assess and juxtapose the image quality and diagnostic performance of accelerated MRI sequences with DL reconstruction against conventional MRI sequences and accelerated MRI without DL in the evaluation of hip joint conditions. This study fills a critical gap in the existing literature, shedding light on the specific utility of DL-based reconstruction in the domain of hip pain assessment.

## Patients and methods

### Participants

This study was approved by the Research Ethics Committee of the First Affiliated Hospital of Zhengzhou University. All study procedures were performed in accordance with the 1975 Declaration of Helsinki, and written informed consent was obtained from all participants before the experiment. It was important to mention that this study also included minor participants under 16 years of age and we had recived informed consent to participate was obtained from the parents or legal guardians of any participant under the age of 16. Our search encompassed electronic medical records to identify patients who had undergone 3-T MRI for hip pain between December 12, 2022, and March 6, 2023. Initially, 63 patients were identified. However, one examination was excluded due to the absence of abnormalities in the patient’s hip joint examination, while two additional examinations were excluded as the patients were postoperative cases with femoral head necrosis and hip implants that could potentially impact image quality.

**Fig. 1:**
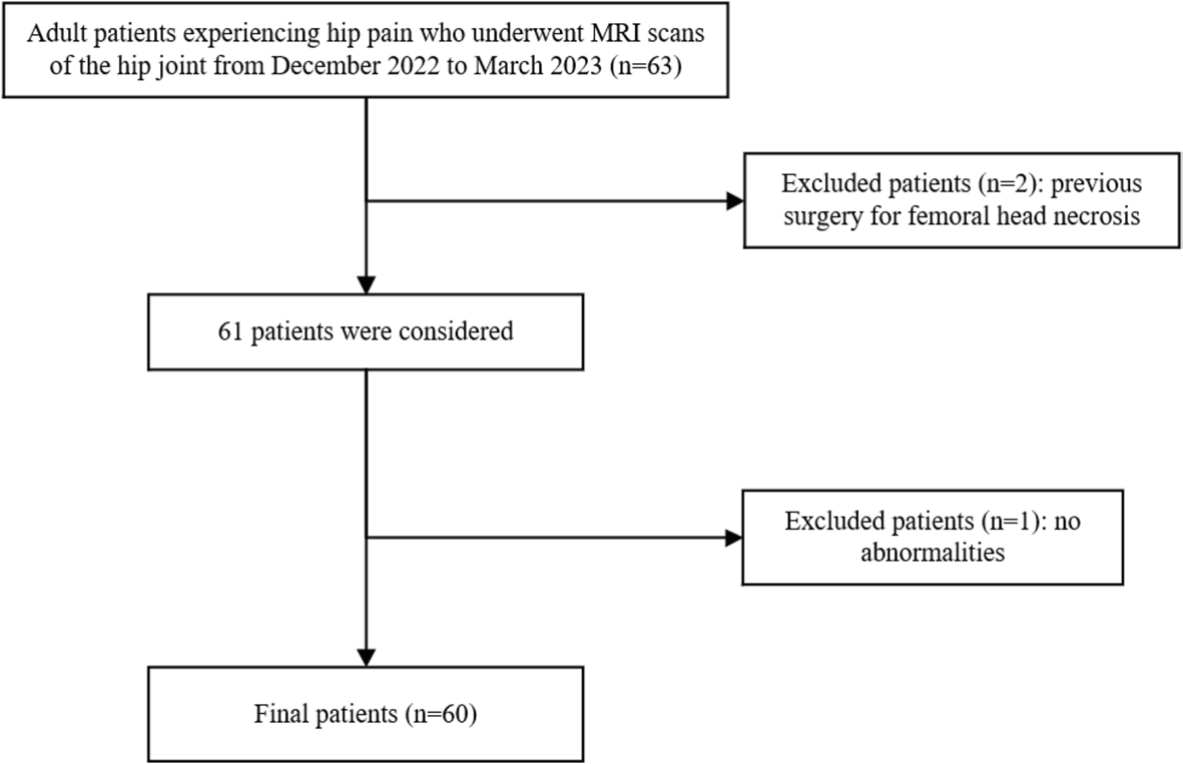
Study flow diagram for patient selection

In this investigation, conventional MRI served as the benchmark for standard MRI scans, No-DL-MRI represented the standard MRI-accelerated sequence, and DL-MRI denoted an MRI-accelerated sequence reconstructed through DL techniques. The study cohort comprised a total of 60 patients, ranging in age from 10 to 65 years, including 29 men and 31 women. A detailed depiction of the study’s patient selection process can be found in Fig. 1.

### MR acquisition

The experiments were conducted using a 3T GE MRI scanner (SIGNATM Premier, GE Healthcare, Waukesha, WI), with patients positioned in the supine orientation and the affected arm placed at their side. Standard clinical MRI protocols for hip evaluation typically encompassed sequences such as coronal T1-weighted imaging (T1WI), coronal T2-weighted imaging (T2WI), coronal fat-saturated (FS) T2WI, axial T2WI, and axial FS T2WI. The total scan time for a conventional hip MRI was approximately 6 min and 56 s. With the implementation of DL reconstruction, the scan duration for standard hip MRI scans could be substantially reduced to as little as 2 min and 32 s. However, given that clinical diagnosis primarily relied on coronal FS T2WI and axial FS T2WI sequences, our analysis primarily focused on these two sequences. The average scan time for the standard hip joint protocol in these two sequences was 3 min and 32 s, in contrast to the accelerated protocol used for DL-based reconstruction, which required only 1 min and 11 s. Detailed parameters for each sequence, encompassing both accelerated MRI and conventional MRI, are provided in Table 1.

**Table 1:** MRI parameters for DL MRI and conventional reconstruction

Parameters	Coronal FS T2WI		Axial FS T2WI	
	DL	Conventional	DL	Conventional
Repetiton time (msec)	2135	2135	2895	2895
Echo time (msec)	50	50	50	50
Field of view (mm^2^)	24 × 24	24 × 24	24 × 24	24 × 24
Slice thickness (mm)	4	4	4	4
Flip angle (degree)	90°	90°	90°	90°
NEX	1	2	1	2
Slices	18	18	24	24
Acceleration factor	3	2	3	2
Acquisition time	30″	1′30″	41″	2′2″

*T2WI* T2-weighted imaging, *fs* fat suppression, *Cor* Coronal, *DL* sequences reconstructed using the vendor-provided Recon DL algorithm, conventional MRI scanning techniques using conventional reconstruction methods, Acceleration factor, pertains to the rapid acquisition technique, namely Autocalibrating Reconstruction for Cartesian sampling, employed during the acquisition process

The DL Recon prototype (GE Healthcare) employed in this study utilized a feed-forward deep CNN-based image reconstruction approach, characterized by enhanced signal-to-noise ratios, diminished truncation artifacts, and heightened spatial resolution [15]. This CNN accepted unfiltered, raw, complex-valued input images and provided noise reduction (NR) at levels customized to the user’s desired output image quality. The improved images demonstrated reduced noise variance at the specified NR level, represented as a percentage ranging from 0 to 100%. The network architecture was rooted in a variant of the residual encoder architecture, a model known for its effectiveness in tasks like super-resolution, image denoising, and JPEG artifact reduction [16]. This DL Recon method was tailored for 2D anatomical sequences and was compatible with a range of standard sequences and options. It delivered substantial enhancements in image quality and sharpness, exhibited minimal truncation artifacts, and showcased robust generalization performance across all anatomical structures. For the purposes of this study, we utilized a 75% NR level.

### Image analysis

MRI images were subject to independent assessment by two readers, each with distinct levels of experience: one board-certified radiologist with 2 years of expertise and another board-certified radiologist boasting over 10 years of specialization in musculoskeletal radiology. These readers were kept blind to any clinical information throughout the evaluation process. All image sets underwent de-identification, ensuring the removal of all sequence identifiers, and were subsequently shuffled into a random order. The revelation of sequence type information occurred only after the initial readouts, serving the purpose of subsequent statistical analysis.

### Qualitative assessment of image quality

The image quality of the conventional, No-DL-MRI, and DL sequences was evaluated separately for various anatomical regions, including bone and cartilage (specifically the femoral head and subchondral bone), acetabular region, and the gluteus maximus muscle. This assessment employed a 5-point Likert scale, where scores corresponded to varying levels of quality: 1—indicating poor, 2—suggesting mild, 3—reflecting moderate, 4—denoting good, and 5—signifying perfect. Prior to commencing the evaluation, readers received explicit instructions on how to assign scores, referencing pre-established image examples that exemplified each grade on the 5-point Likert scale. Comparisons were made between conventional MRI and DL-MRI, as well as between No-DL-MRI and DL-MRI. Both sets of comparisons were factored into the overall assessment of image quality.

### Quantitative assessment of the image quality

To quantitatively gauge image quality, we measured the relative signal-to-noise ratio (rSNR) and the relative contrast-to-noise ratio (rCNR) for both MRI sequences. This involved placing circular regions of interest (ROIs) with an area of 60 mm² on distinct anatomical regions, specifically the femoral head, subchondral bone, acetabular region, and the gluteus maximus muscle. The ROIs were meticulously positioned on both regular and DL images to determine the signal intensity (SI) in each of these regions. Three levels showcasing the optimal tissue structures were chosen for delineating the ROIs. Additional details concerning the calculation process can be found in the supplementary material.

### Diagnostic performance

For evaluating interreader and intermethod agreements, both readers assessed pathological lesions, including the degree of femoral head deformation (graded as 1 = absent, 2 = mild, 3 = moderate or severe), the continuity of subchondral bone (graded as 1 = absent, 2 = mild discontinuity, 3 = moderate or severe discontinuity), and stenosis of the articular space (graded as 1 = absent, 2 = mild stenosis, 3 = moderate or severe stenosis) in both Conventional MRI and DL-MRI.

Two readers independently assessed these lesions in a blinded and randomized manner. We determined interobserver agreements by comparing assessments between the two readers, each possessing 2 and 10 years of experience in musculoskeletal radiology, respectively.

### Statistical analysis

Statistical analysis was conducted utilizing SPSS software (version 26.0). To assess the significance of differences in image quality between DL-MRI and conventional MRI, as well as between DL-MRI and No-DL-MRI, and to calculate rSNR and rCNR between conventional MRI and DL-MRI, we employed the Wilcoxon signed-rank test. Interreader agreements were evaluated using the weighted kappa coefficient, with values interpreted as follows: κ = 0 (no agreement), 0 < κ ≤ 0.2 (slight agreement), 0.2 < κ ≤ 0.4 (fair agreement), 0.4 < κ ≤ 0.6 (moderate agreement), 0.6 < κ ≤ 0.8 (substantial agreement), and 0.8 < κ ≤ 1 (almost perfect agreement).

## Results

### Qualitative image quality

The comparative analysis of overall image quality and interobserver agreement scores for both the DL-MRI versus conventional MRI and DL-MRI versus No-DL-MRI groups is presented comprehensively in Table 2.

**Table 2:** Results of subjective image quality scores

Sequences	Reader	Conventional MRI	NO-DL MRI	DL-MRI	*P* (conventional vs. DL)	*P* (no-DL vs. DL)	k of Conventi-onal MRI	k of no-DL-MRI	k of DL-MRI
T2WI-FS-Cor	1	3.47 ± 0.503	2.85 ± 0.515	4.62 ± 0.524	< 0.001*	< 0.001*	0.866	0.735	0.863
	2	3.47 ± 0.503	2.93 ± 0.482	4.65 ± 0.515	< 0.001*	< 0.001*			
T2WI-FS-Axi	1	3.40 ± 0.527	2.93 ± 0.516	4.67 ± 0.510	< 0.001*	< 0.001*	0.774	0.823	0.964
	2	3.45 ± 0.534	2.95 ± 0.534	4.68 ± 0.504	< 0.001*	< 0.001*			

Note: Comparisons were conducted using the paired Wilcoxon signed-rank test. The weighted kappa coefficient was utilized to assess reader agreement. *T2WI *T2-weighted imaging, *FS *Fat Suppression, *Cor *Coronal, *Axi *Axial, DL-MRI images were reconstructed using the vendor-provided DL reconstruction pipeline; *indicates statistically significant differences

For the comparison between DL-MRI and Conventional MRI, subjective image quality scores were obtained from readers 1 and 2. For the accelerated sequence with DLR of coronal FS T2WI, reader 1 assigned a score of 4.62 ± 0.524, while reader 2 rated it at 4.65 ± 0.515. Similarly, for the accelerated sequence with DLR of axial FS T2WI, reader 1 scored it at 4.67 ± 0.510, and reader 2 gave it a score of 4.68 ± 0.504. In contrast, the subjective image quality scores for the conventional MRI sequences of coronal FS T2WI were 3.47 ± 0.503 and 3.47 ± 0.503 for readers 1 and 2, respectively, while for axial FS T2WI, they were 3.40 ± 0.527 and 3.45 ± 0.534. These findings indicate that the overall image quality of DLR was significantly superior to that of conventional MRI, with a statistically significant difference (*P* < 0.001). Notably, substantial interobserver agreement was found for image quality in Conventional MRI (kappa value = 0.774 to 0.866) for both coronal FS T2WI and axial FS T2WI. As for DL-MRI, almost perfect agreement was achieved in coronal FS T2WI (kappa value = 863) and axial FS T2WI (kappa value = 0.964).

Turning our attention to the comparison between DL-MRI and No-DL-MRI, the subjective image quality scores for readers 1 and 2 aligned consistently with the results from the accelerated sequence with DLR. Specifically, for the No-DL-MRI sequence of coronal FS T2WI, reader 1 assigned a score of 2.85 ± 0.515, and reader 2 rated it at 2.93 ± 0.482. For the No-DL-MRI sequence of axial FS T2WI, reader 1 assigned a score of 2.93 ± 0.516, while reader 2 rated it at 2.95 ± 0.534. Once again, the difference in image quality between DL-MRI and No-DL-MRI was statistically significant (*P* < 0.001). Substantial agreement for image quality was achieved for DL-MRI (kappa value = 0.735) in coronal FS T2WI, and almost perfect agreement was observed in T2WI FS Axi (kappa value = 0.823). Additionally, for No-DL-MRI, there was almost perfect agreement in both coronal FS T2WI (kappa value = 0.863) and axial FS T2WI (kappa value = 0.964).

**Fig. 2:**
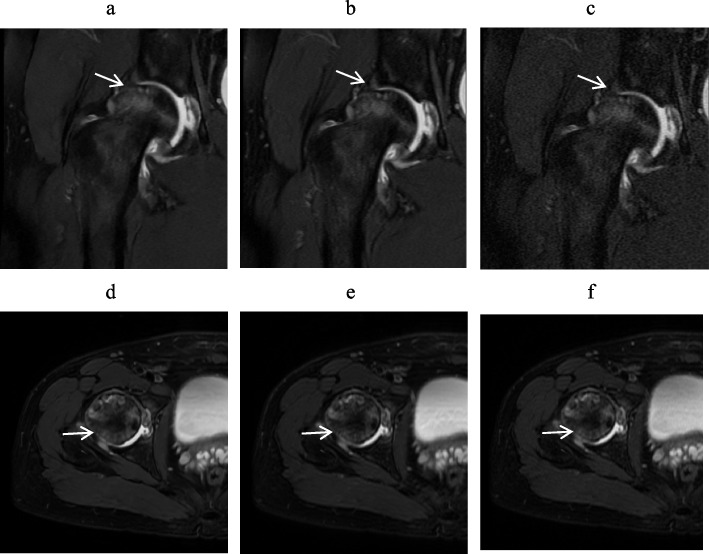
A 42-year-old man with hip pain underwent a hip MRI. **a**, **d** Coronal (Cor) and Axial (Axi) Fat Suppressed T2-weighted Imaging Using Deep Learning Reconstruction. **b**, **e** Standard MRI Images Employing Conventional Reconstruction Methods. **c**, **f** Accelerated Sequences with Conventional Reconstruction. The white arrow indicates the location of interest. DL images exhibited superior overall image quality and lower noise levels compared to conventional MRI and accelerated sequences.

Figure 2 provides a visual representation of the differences in overall image quality among DL-MRI, conventional MRI, and No-DL-MRI. A clear comparison reveals that the image quality of both conventional MRI and No-DL-MRI is inferior to that of DL-MRI, characterized by reduced noise and higher overall image quality.

### Comparison of rSNR and rCNR

Table 3 presents the results of rSNR for various anatomical structures in DL-MRI and No-DL-MRI across different sequences. Furthermore, Table 4 illustrates the rCNR between the first three tissues and the gluteus maximus in DL-MRI and No-DL-MRI across different sequences.

**Table 3:** Results of rSNR for different anatomical structure in DL-MRI and No-DL-MRI across different sequences

Sequences	organization	No-DL-MRI	DL-MRI	z	*P*
coronal FS T2WI	femoral head	0.5683(0.4350,0.7025)	0.8000(0.7500,0.8700)	−6.361	<0.001*
	subchondral bone	0.7783(0.6917,0.8558)	0.8183(0.7900,0.8875)	−4.703	<0.001*
	acetabular	0.6066(0.5250,0.6767)	0.6000(0.5700,0.6392)	−0.011	0.991
	gluteus maximus	0.3950(0.2950,0.6642)	0.8233(0.7350,0.8900)	−6.515	<0.001*
axial FS T2WI	femoral head	0.3983(0.3108,0.6092)	0.7916(0.7000,0.8658)	−6.383	<0.001*
	subchondral bone	0.7200(0.6675,0.7733)	0.7966(0.7542,0.8625)	−5.192	<0.001*
	acetabular	0.7750(0.6492,0.8383)	0.7516(0.6517,0.7517)	−0.195	0.845
	gluteus maximus	0.4533(0.3308,0.6033)	0.7200(0.6308,0.8192)	−6.401	<0.001*

*rSNR *Relative Signal-to-Noise Ratio, No-DL-MRI - Conventional MRI with Accelerated Sequences, *DL MRI* Accelerated MRI Sequences Reconstructed Using the Vendor-Provided Recon DL Algorithm, *FS *Fat Suppression, *Cor *Coronal, *Axi *Axial, *T2WI* - T2-Weighted Imaging. *P* values denoted with an asterisk indicate statistical significance

**Table 4:** Results of rCNR for different anatomical structure relative to gluteus maximus in DL-MRI and No-DL-MRI across different sequences

Sequences	organization	No-DL-MRI	DL-MRI	z	*P*
coronal FS T2WI (vs. gluteus maximus)	the femoral head	0.3816(0.2742,0.5883)	0.7716(0.6567,0.8392)	−5.69	<0.001*
	subchondral bone	0.4633(0.3575,0.6042)	0.6900(0.5567,0.7867)	−5.797	<0.001*
	Acetabular	0.4383(0.3375,0.6467)	0.6650(0.5258,0.7917)	−3.616	<0.001*
axial FS T2WI (vs. gluteus maximus)	the femoral head	0.5266(0.4075,0.6250)	0.7647(0.6408,0.8491)	−5.838	<0.001*
	subchondral bone	0.5450(0.4175,0.6475)	0.5200(0.4341,0.6844)	−0.85	0.395
	Acetabular	0.5133(0.3800,0.7000)	0.5070(0.4337,0.5809)	−0.427	0.669

*rCNR *Relative Contrast-to-Noise Ratio, No-DL-MRI - Conventional MRI with Accelerated Sequences, *DL MRI *Accelerated MRI Sequences Reconstructed Using the Vendor-Provided Recon DL Algorithm. *FS *Fat Suppression, *T2WI *T2-Weighted Imaging

*P* values denoted with an asterisk indicate statistical significance

Since the data for rSNR and rCNR did not follow a normal distribution, we employed a paired Wilcoxon test, and the results are presented as medians with upper and lower quartiles. The table clearly demonstrates that, with the exception of the acetabular region, the majority of rSNR data points were significantly higher in coronal FS T2WI and axial FS T2WI of DL-MRI compared to No-DL-MRI (*p* < 0.001). Similarly, for rCNR, the data were also significantly higher in coronal FS T2WI of DL-MRI (*p* < 0.001). However, the results for the acetabular region differed from the others, with no significant differences observed in coronal FS T2WI (*P* = 0.991) and axial FS T2WI (*P* = 0.845). This outcome may be attributed to the small size and low SNR of the iliac socket, rendering it susceptible to factors such as joint cavity effusion. It also suggests that DLR may need further optimization to address low-SNR areas. Moreover, not all rCNR results were significantly different in axial FS T2WI (*P* = 0.395). Specifically, the rCNR of the subchondral bone and acetabular regions showed no significant differences in axial FS T2WI (*P* = 0669).

**Fig. 3:**
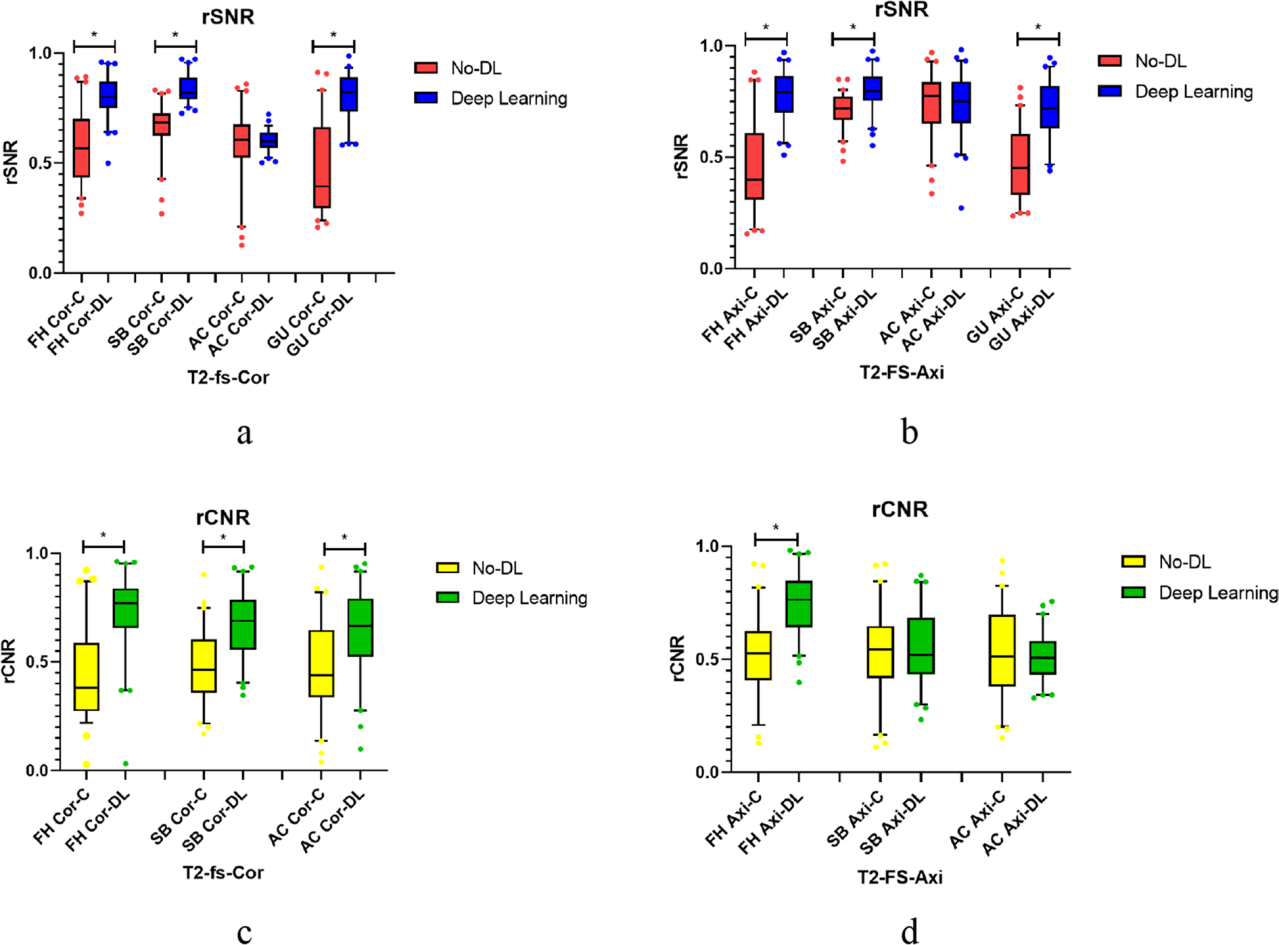
Comparison of rSNR and rCNR in DL-MRI vs. No-DL-MRI. **a**Comparison of rSNR for Different Anatomical Structures in T2 FS Coronal Sequences. **b** Comparison of rSNR for Different Anatomical Structures in T2 FS Axial Sequences. **c** Comparison of rCNR between Different Anatomical Structures and Gluteus Maximus in T2 FS Coronal Sequences. **d** Comparison of rCNR between Different Anatomical Structures and Gluteus Maximus in T2 FS Axial Sequences. DL MRI represents Accelerated MRI Sequences Reconstructed Using the Vendor-Provided Recon DL Algorithm. No-DL-MRI signifies Conventional MRI with Accelerated Sequences. FS - Fat Suppression; Cor - Coronal; Axi - Axial; T2WI - T2-Weighted Imaging. Anatomical structures include FH (Femoral Head), AC (Acetabular), SB (Subchondral Bone), and GU (Gluteus Maximus)

While isolated data points indicated no significant difference between DLR and No-DL-MRI, the majority of results demonstrated that accelerated sequences reconstructed using DLR exhibited higher rSNR and rCNR, as depicted in Fig. 3.

### Diagnostic performance

Table 5 provides an overview of the diagnostic evaluation conducted by two observers regarding femoral head deformation, subchondral bone continuity, and articular space stenosis in DL-MRI and conventional MRI.

**Table 5:** Results of diagnostic performance

	Reader		Numbers of patients with scores of 3/2/1			Comparison *p* values	Interobsever agreement(k)	
				DL-MRI	Conventional MRI		DL-MRI	Conventional MRI
coronal FS T2WI	deformation of the femoral head	1		3/10/47	1/12/47	0.157	0.962	0.916
		2		3/11/46	2/12/46	0.014*		
	continuity of subchondral bone	1		1/12/47	1/11/48	0.317	0.830	0.765
		2		1/14/45	3/13/44	0.317		
	stenosis of the articular space	1		1/16/43	2/13/45	0.317	0.736	0.682
		2		2/22/36	3/14/43	0.005*		
axial FS T2WI	deformation of the femoral head	1		3/10/47	1/14/45	0.564	0.812	0.875
		2		3/11/46	1/13/46	0.157		
	continuity of subchondral bone	1		1/13/46	0/12/48	0.020*	0.920	0.904
		2		2/13/45	1/12/47	0.655		
	stenosis of the articular space	1		2/18/40	1/14/45	0.157	0.845	0.689
		2		2/23/35	3/17/40	0.025*		

Comparisons were conducted using the Paired Wilcoxon signed-rank test. Interobserver agreement is measured using the weighted kappa coefficient between two readers. Conventional MRI denotes conventional MRI sequences reconstructed using traditional methods. DL MRI represents accelerated MRI sequences reconstructed using the vendor-provided Recon DL algorithm. *FS *Fat Suppression, *T2WI *T2-Weighted Imaging. Statistical significance is indicated by an asterisk

For the diagnosis of femoral head deformation in coronal FS T2WI, there was perfect agreement between the two observers in both DL MRI (kappa value = 0.962) and conventional MRI (kappa value = 0.916). Notably, it is worth mentioning that observer 2’s ratings differed significantly between the two methods (*P* = 0.014), while observer 1 did not exhibit such a difference (*P* = 0.157). In assessing the continuity of subchondral bone, there was almost perfect interobserver agreement, indicated by the kappa value of 0.830 for both DL MRI and 0.765 for conventional MRI. Moreover, inter-reader agreement after DLR was higher compared to conventional MRI. Regarding the diagnosis of articular space stenosis, substantial interobserver agreement was observed for both DL-MRI (kappa value = 0.736) and conventional MRI (kappa value = 0.682). It is noteworthy that observer 1’s ratings showed a significant difference between the two methods (*P* = 0.005), whereas observer 2 did not exhibit such a difference (*P* = 0.317).

In axial FS T2WI, we observed almost perfect agreement regarding femoral head deformation, with the kappa value of 0.812 for both DL-MRI and 0.875 for conventional MRI. This consensus was consistent for both observer 1 (*P* = 0.564) and observer 2 (*P* = 0.157). For the assessment of subchondral bone continuity, perfect interobserver agreement was observed for both DL-MRI (kappa value = 0.920) and conventional MRI (kappa value = 0.904). However, it’s important to note that observer 2’s ratings showed a significant difference between the two methods (*P* = 0.020), whereas observer 1 did not exhibit such a difference (*P* = 0.655). In the diagnosis of articular space stenosis, we achieved almost agreement for DL-MRI (kappa value = 0.845). The substantial agreement for stenosis of the articular space was achieved of DL-MRI (kappa value = 0.689). It is noteworthy that observer 1’s ratings showed a significant difference between the two methods (*P* = 0.025), while observer 2 did not exhibit such a difference (*P* = 0.157).

**Fig. 4:**
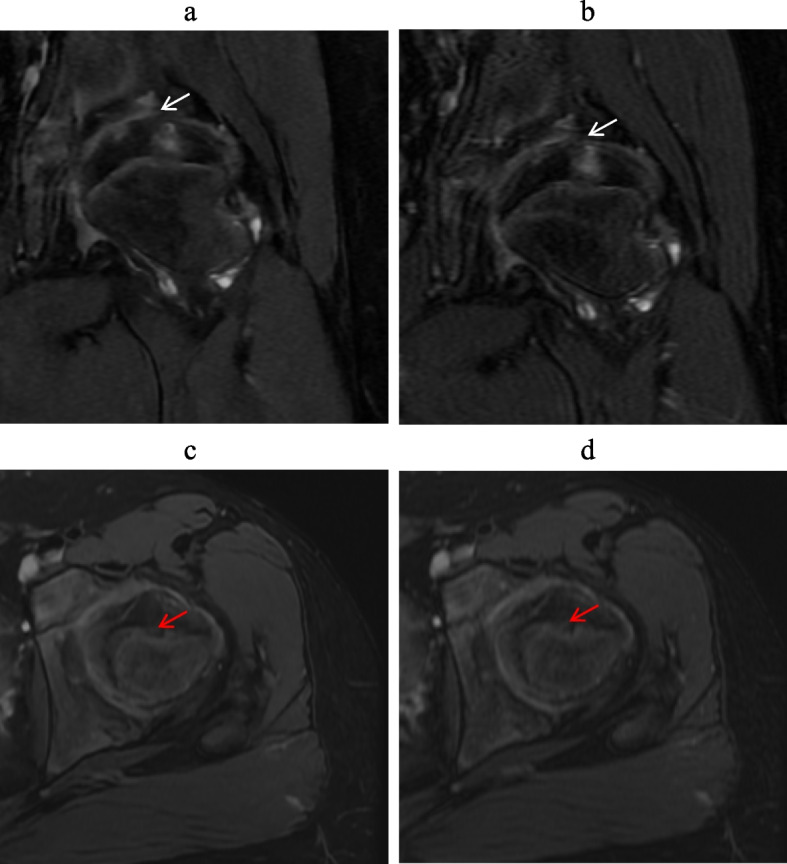
A 11-year-old man with hip pain underwent a hip MRI. Images include coronal (Cor) and axial (Tra) fat-suppressed T2-weighted imaging based on deep learning reconstruction (**a**, **c**), and standard MRI images using conventional reconstruction methods (**b**, **d**). When assessing femoral head deformation (indicated by the red arrow), both readers assigned a score of 2. For evaluating subchondral bone continuity, both readers also assigned a score of 2. In the assessment of articular space stenosis (indicated by the white arrow), reader 1 assigned a score of 2, while reader 2 assigned a score of 3. These results suggest that DL-MRI and conventional MRI exhibit similar diagnostic capabilities

**Fig. 5:**
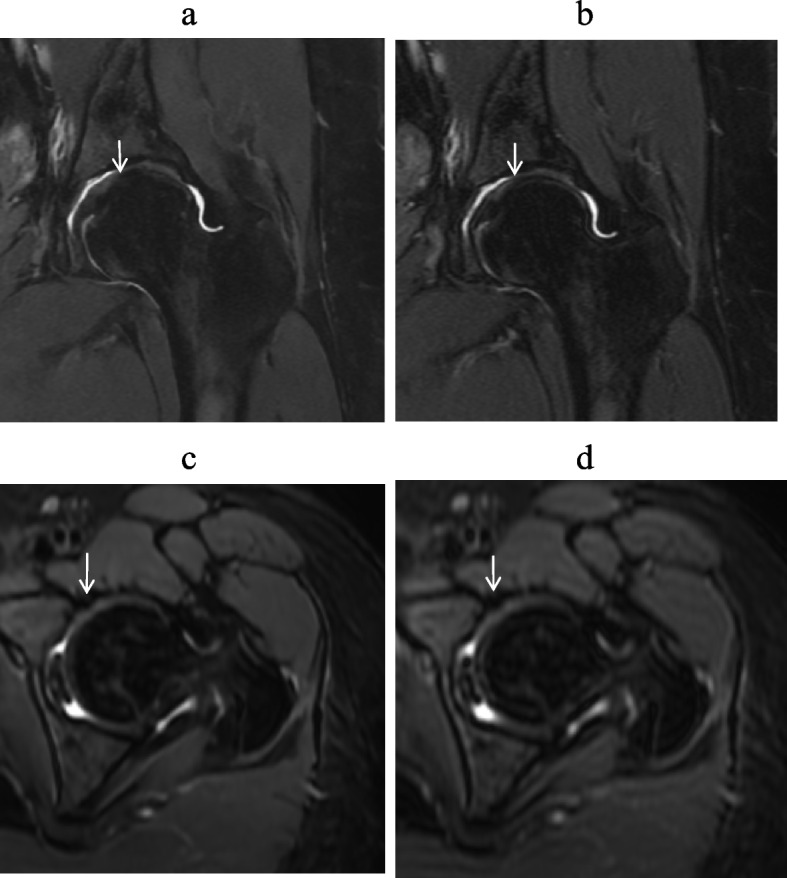
A 34-year-old woman with hip pain underwent a hip MRI. Images consist of coronal (Cor) and axial (Tra) fat-suppressed T2-weighted imaging based on deep learning reconstruction (**a**, **c**), and standard MRI images using conventional reconstruction methods (**b**, **d**). Both readers assigned a score of 1 for femoral head deformation assessment. In evaluating subchondral bone continuity, reader 1 assigned a score of 1, while reader 2 assigned a score of 2. Additionally, both readers assigned a score of 1 when assessing articular space stenosis (indicated by the arrow). These results also indicate that DL-MRI and conventional MRI yield similar diagnostic performance

**Fig. 6:**
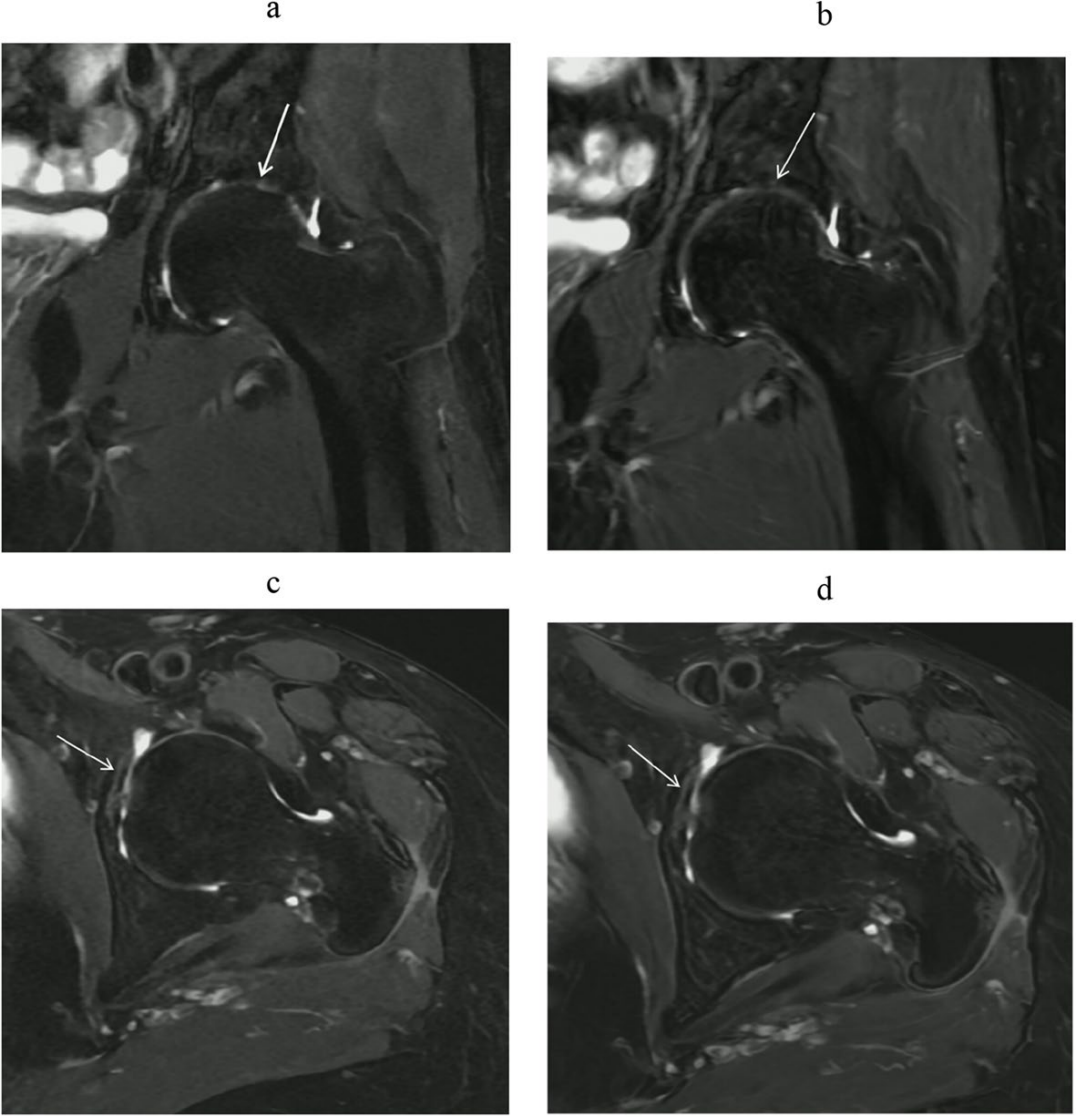
A 65-year-old woman with hip pain underwent a hip MRI. Images consist of coronal (Cor) and axial (Tra) fat-suppressed T2-weighted imaging based on deep learning reconstruction (**a**, **c**), and standard MRI images using conventional reconstruction methods (**b**, **d**). Both readers assigned a score of 1 for femoral head deformation assessment. In evaluating subchondral bone continuity, both readers assigned a score of 1. Additionally, both readers assigned a score of 1 when assessing articular space stenosis (indicated by the arrow). These results also indicate that DL-MRI and conventional MRI yield similar diagnostic performance

These results underscore the presence of diagnostic bias influenced by the seniority and experience of the radiologists when interpreting different images. However, DL demonstrated superior inter-reader agreement compared to conventional images. This observation is further supported by the clear illustration of diagnostic performance in both DL-MRI and conventional MRI in Figs. 4, 5 and 6.

## Discussion

In our study, our results unequivocally demonstrated the remarkable capability of the DL method to accelerate hip joint MR imaging, reducing scan time by approximately 66.5% when compared to conventional MRI. The integration of DLR with accelerated sequences yielded notable enhancements in image quality compared to accelerated sequences devoid of DLR and traditional MRI. Notably, DL-MRI excelled in achieving higher rSNR and rCNR in coronal FS T2WI and axial FS T2WI sequences in comparison to No-DL-MRI. These findings highlight the pivotal role played by DLR in facilitating the practical application of accelerated sequences in clinical hip joint MRI.

In the realm of accelerated imaging, parallel imaging has long held a prominent position, especially in joint imaging, owing to its accessibility [17]. Nevertheless, it is essential to acknowledge the inherent limitations of parallel imaging, including a reduction in Signal-to-Noise Ratio (SNR) and the emergence of acceleration artifacts. While these limitations can be partially mitigated through the use of higher-field-strength magnets and multi-channel coils in clinical practice, a more transformative solution has emerged in the form of DLR. As anticipated, both our study and previous research have consistently demonstrated the superior image quality and quantitative measurements achieved by DLR. For instance, a study conducted by Kevin M et al. [13], which compared relative anatomic edge sharpness, rSNR, and rCNR of two-dimensional fast spin-echo MRI with DL models applied at 50% and 75% NR settings for hip and shoulder imaging, revealed that DL reconstruction significantly improved edge sharpness, rSNR, and rCNR when compared to conventional reconstructions. Similar findings were reported by Seok et al. [14], who observed comparable sensitivity and specificity of shoulder imaging in terms of subjective image quality, artifacts, and diagnostic performance when compared to standard sequences. Additionally, Geojeong et al. [18] demonstrated that DLR sequences substantially reduced acquisition time for cervical spine imaging using the Dixon sequence, with subjective image quality and lesion detectability at least on par with conventional sequences. Thomas Dratsch et al. [19] combined compressed perception with DL reconstruction techniques for accelerating three-dimensional (3D) magnetic resonance imaging (MRI) sequences of the knee. The findings demonstrate that images reconstructed using CS-AI consistently achieved significantly higher ratings for subjective measurements of image quality across all acceleration levels, compared to the corresponding images reconstructed using CS. Importantly, sequences reconstructed using DL yielded higher rSNR and rCNR in our research. And these robust agreement scores suggest that DL-MRI, when used for hip MRI sequences, maintains diagnostic accuracy consistently across both experienced and inexperienced observers. This not only holds potential for reducing patient scan times but also for enhancing overall clinical efficiency.

The utilization of DL image reconstruction is becoming increasingly pervasive across various medical imaging domains, with a primary objective of NR to ensure superior image quality while simultaneously accelerating scan speed [20]. Chen et al. illustrated the immense potential of CNN-based reconstruction techniques in effectively suppressing image noise, preserving structural integrity, and detecting lesions [21]. The work of Schlemper et al. underscored the utility of CNN-based methods in the reconstruction of 2D cardiac MRI, showcasing reduced reconstruction errors and expedited reconstruction speeds [22]. Collectively, these prior investigations have underscored the pivotal role of DLR in expediting MRI scans. It is noteworthy that our study aligns with these findings, affirming that DL-MRI can expedite T2WI scans and concurrently enhance image quality in hip imaging.

Our research unveiled that DL-MRI not only reduces scanning time but also upholds diagnostic performance. In patients presenting with hip pain, the susceptibility to motion artifacts stemming from the patients’ movements introduces varying degrees of diagnostic complexity. Significantly, reducing scan time holds the potential to mitigate motion artifacts [23]. Our results thus substantiate the pivotal role of DL-MRI in catalyzing the adoption of accelerated sequences as a viable alternative to conventional sequences, with the overarching aim of minimizing motion-induced artifacts in hip imaging.

### Limitations and prospects

This study, while insightful, possesses certain limitations that warrant acknowledgment. Primarily, its sample size was relatively small, suggesting that future investigations should consider expanding the cohort to encompass a broader demographic spectrum. Additionally, our study exclusively focused on the coronal FS T2WI and axial FS T2WI sequences, driven by their clinical relevance at the time of diagnosis. However, future research endeavors should encompass a more extensive array of MRI sequences to elucidate the broader applicability of DLR techniques across diverse imaging contexts.

## Conclusions

In summary, our findings underscore the remarkable potential of DL-MRI in revolutionizing hip imaging. This innovative approach not only expedites scan times but also significantly enhances imaging quality. While variations in lesion detectability between Conventional MRI and DL-MRI were noted among different readers, it is noteworthy that inter-observer consistency showed an overall improvement. These compelling outcomes not only reinforce the clinical viability of DL-MRI but also portend exciting prospects for its widespread adoption in the realm of medical imaging.

## Supplementary Information

Additional file 1.

## Data Availability

The datasets used and/or analysed during the current study are available from the corresponding author on reasonable request.

## Abbreviations

DL: Deep learning
DLR: Deep learning based reconstruction
DL-MRI: Deep learning based reconstruction Magnetic Resonance Imaging
No-DL-MRI: Accelerated imaging without deep learning
rSNR: Relative Signal-to-Noise Ratio
rCNR: Relative Contrast-to-Noise Ratio
FS: fat-saturated
MRI: Magnetic Resonance Imaging
CNN: Convolutional neural network
T1WI: T1-weighted imaging
T2WI: T2-weighted imaging
NR: noise reduction
ROIs: regions of interest
SI: signal intensity
SNR: Signal-to-Noise Ratio
