# Supplementary material for: Optimizing hip MRI: enhancing image quality and elevating inter-observer consistency using deep learning-powered reconstruction
Source: BMC Med Imaging. 2025 Jan 13;25:17. doi: 10.1186/s12880-025-01554-y (PMC11730829; doi:10.1186/s12880-025-01554-y)
Supplement: Supplementary file 1 — Additional file 1. [file 12880_2025_1554_MOESM1_ESM.docx]

Supplementary material:

To quantitatively assess image quality, the relative signal-to-noise ratio (rSNR) and the relative contrast-to-noise ratio (rCNR) for both sequences were measured. Circular regions of interest (ROIs) of 60 mm2 were placed separately on each set to define the signal intensity (SI) in bone (in the femoral head), subchondral bone, acetabular and muscle (gluteus maximus). And the three levels that showed the best tissue structure were selected to sketch ROI on both regular and DL images.

The following methods were used for each relative metric:

Relative Signal to Noise Ratio and Relative Contrast to Noise Ratio:

$$rSNR=\max Norm\left(\frac{S}{STD}\right)$$

$$rCNR=\mathrm{maxNorm}\left(\frac{S_{\text{muscle }}-S_{\text{bone }}}{\mathrm{mean}\left(STD_{\text{bone }},STD_{\text{muscle }}\right)}\right)$$

where S was the signal of the given ROI, STD was the standard deviation within the ROI, and

$$\max Norm(f_{\text{j}})=\frac{f_{j}}{\max(\forall_{i}f_{i})}$$
